# Integrating long-acting injectable treatment to improve medication adherence among persons living with HIV and opioid use disorder: study protocol

**DOI:** 10.1186/s13722-023-00418-6

**Published:** 2023-10-14

**Authors:** Kirsten J. Langdon, Anthony E. Hitch, Alexandra B. Collins, Curt G. Beckwith, Sara Becker, Karen Tashima, Josiah D. Rich

**Affiliations:** 1 Department of Psychiatry, Rhode Island Hospital, 139 Point Street, Providence, RI 02903 USA; 2 Department of Psychiatry and Human Behavior, Alpert Medical School of Brown University, Providence, USA; 3 Brown-Lifespan Center for Digital Health, Providence, USA; 4 Department of Psychology, University of Cincinnati, Cincinnati, USA; 5 Department of Epidemiology, Brown University School of Public Health, Providence, USA; 6 Center for Dissemination and Implementation Science, Northwestern University, Evanston, USA; 7 Department of Medicine, Division of Infectious Diseases, Brown University and The Miriam and Rhode Island Hospitals, Providence, USA

**Keywords:** Opioid use disorder, HIV, Medications for opioid use disorder, Long-acting injectable medication, Antiretroviral therapy, Antiviral resistance

## Abstract

**Background:**

Oral antiretroviral therapy (ART) has been effective at reducing mortality rates of people with HIV. However, despite its effectiveness, people who use drugs face barriers to maintaining ART adherence. Receipt of opioid agonist treatment, in the context of HIV care, is associated with medication adherence and decreased HIV viral loads. Recent pharmacological advancements have led to the development of novel long-acting, injectable, medications for both HIV (cabotegravir co-administered with rilpivirine) and OUD (extended-release buprenorphine). These therapies have the potential to dramatically improve adherence by eliminating the need for daily pill-taking. Despite the extensive evidence base supporting long-acting injectable medications for both HIV and OUD, and clinical guidelines supporting integrated care provision, currently little is known about how these medications may be optimally delivered to this population. This paper presents the study design for the development of a clinical protocol to guide the delivery of combined treatment for HIV and OUD using long-acting injectable medications.

**Methods:**

The study aims are to: (1) develop a clinical protocol to guide the delivery of combined LAI for HIV and OUD by conducting in-depth interviews with prospective patients, clinical content experts, and other key stakeholders; and (2) conduct This single group, open pilot trial protocol to assess feasibility, acceptability, and safety among patients diagnosed with HIV and OUD. Throughout all phases of the study, information on patient-, provider-, and organizational-level variables will be collected to inform future implementation.

**Discussion:**

Findings from this study will inform the development of a future study to conduct a fully-powered Hybrid Type 1 Effectiveness-Implementation design.

## Background

The overdose epidemic continues to be a major public health crisis in the United States, with rates of opioid-involved fatal and non-fatal overdoses steadily rising [1, 2]. Persons with HIV (PWH) who use drugs are especially vulnerable to fatal overdose [3]. Moreover, untreated opioid use disorder (OUD) is associated with higher rates of HIV infection and transmission [4, 5]. Despite a decline in new HIV infections from 2000 to 2015, regions across the United States have documented an increase in isolated HIV outbreaks among people who inject drugs [6]. The COVID-19 pandemic has further complicated this issue as rates of injection drug use, as well as fatal and non-fatal overdoses, have continued to rise [1]. Such data highlight the complex association between HIV and OUD, and the significant societal impact of these intertwined epidemics [7].

Oral antiretroviral therapy (ART) has been effective at reducing mortality rates of PWH, including PWH who use drugs. However, despite its effectiveness, people who use drugs often face barriers to maintaining ART adherence, thereby increasing their risk of HIV disease progression, a shortened lifespan, greater risk of HIV transmission, and the development of treatment resistant strains of HIV [8–14]. Relative to those who do not use drugs, PWH who use drugs are more than twice as likely to face social and structural inequities (e.g., housing instability, insurance barriers) impacting their adherence to ART, thereby contributing to increased morbidity, mortality, and onward HIV transmission [8–10, 15–17].

**Fig. 1:**
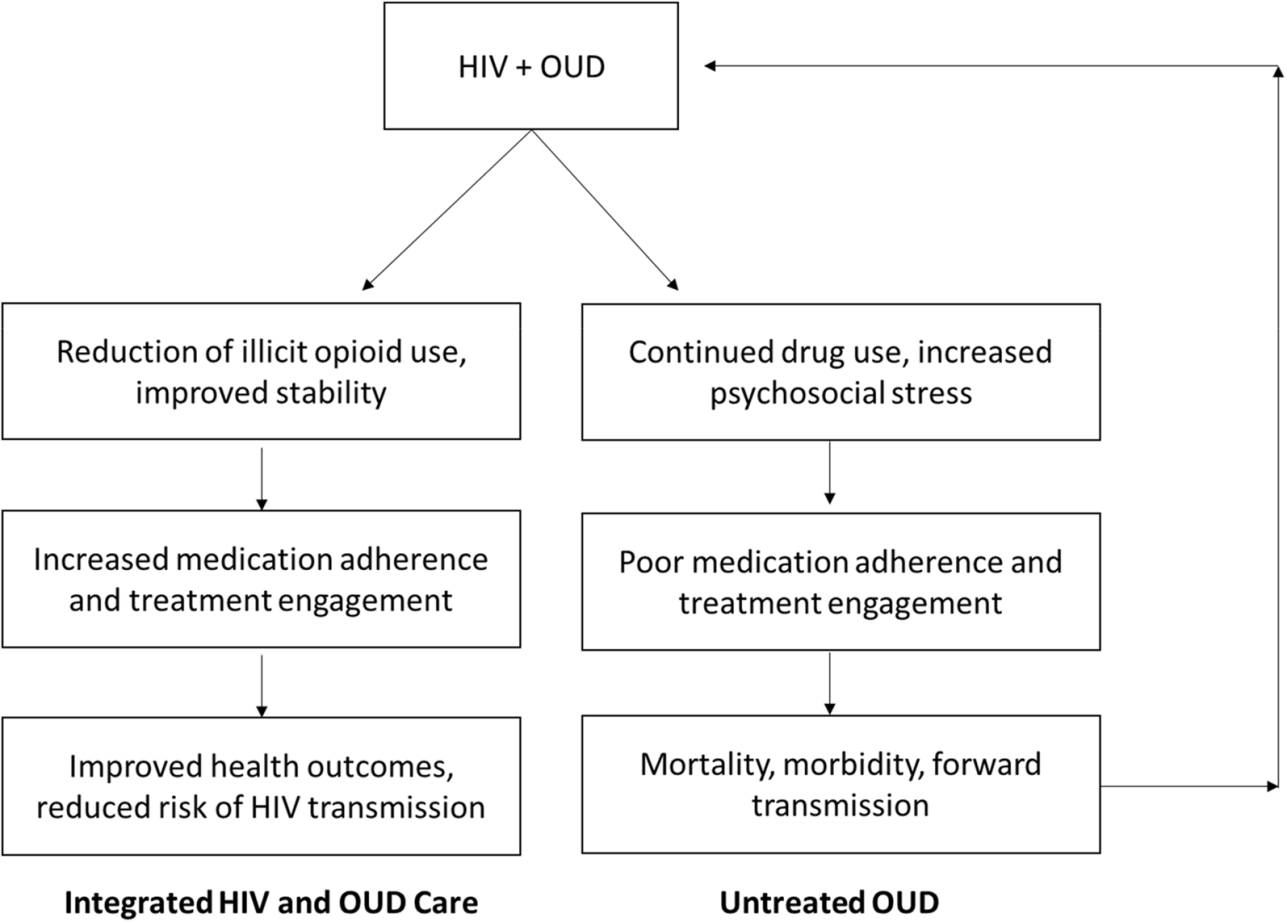
Conceptualization of integrated treatment for HIV and OUD

Treatment services for HIV and OUD have historically been delivered across multiple settings leading to fragmented and uncoordinated care. Along the continuum of care, people who use drugs frequently experience substantial disruptions, including delayed entry into HIV care [18, 19], suboptimal initiation of ART during advanced stages of disease [20], and discontinuation of ART [21, 22]. Models of behavior change suggest that addressing multiple chronic conditions simultaneously through integrated, evidence-based interventions can have a synergistic effect resulting in improved health-related outcomes [23–25]. Consistent with this perspective, numerous studies have documented that receipt of medications for OUD in the context of HIV care is associated with treatment retention, ART adherence, and HIV viral suppression [26–29]. In light of the Ending the HIV Epidemic campaign to reduce new HIV infections in the United States by 90% by 2030 [30], there have been calls to develop integrated or co-located HIV and OUD services to expand access to care [31–34]. Integrated treatment models that improve adherence to HIV and OUD care are critical to prevent the continued spread of HIV in the context of the current overdose crisis (see Fig. 1).

Recent advances in pharmacological treatment have led to the development of novel long-acting injectable (LAI) medications for HIV (cabotegravir co-administered with rilpivirine; CAB/RPV) and OUD (extended-release buprenorphine; XR-B). These therapies have the potential to improve adherence significantly, lead to better control of both diseases, and reduce mortality rates for PWH who use drugs. Further, LAI ART may have an advantage over daily oral ART in preventing sub-therapeutic drug levels resulting from missed oral doses that can lead to HIV drug resistance [35–37]. However, missed LAI ART injections or discontinuation of LAI ART without prompt initiation of oral ART also carries the risk of developing ART resistance. The goal of LAI ART is to have a multi-drug regimen that is delivered by intramuscular injection, with reliable pharmacokinetic properties to allow for infrequent dosing (currently approved for monthly or bi-monthly dosing), and to achieve comparable potency and efficacy to oral ART with similar side effect profiles [38]. Clinical trial data demonstrate non-inferiority of monthly injections of ART compared to the ‘gold standard’ oral daily ART [39]. Further, more than 90% of participants who received LAI in randomized clinical trials preferred monthly injectable therapy over daily oral therapy [40]. For patients with OUD, XR-B is a once-monthly buprenorphine injection designed to deliver therapeutic plasma concentrations for the treatment of moderate to severe OUD [41]. XR-B has demonstrated efficacy and safety in the community [42, 43]. Compared to sublingual forms of buprenorphine, XR-B offers potential feasibility benefits including reduced likelihood of diversion and improved medication adherence to avoid illicit opioid use and overdose.

Despite the potential of integrating LAI medications for HIV and OUD, there are substantial gaps in knowledge about how to best co-deliver treatment. First, in the LAI ART trials, individuals with an active substance use disorder were excluded from participation, thus, the experiences of those with an OUD were not represented. Second, past research has yet to fully evaluate: (a) perceived acceptability/feasibility of combining LAI medications for HIV and OUD into a single point of care; (b) the safety of co-administering LAI medications for HIV and OUD; (c) which populations and in what type of settings these medications may be optimally delivered; and (d) what factors may impede or facilitate future implementation of co-locating these treatments. This study protocol fills existing research gaps by conducting formative research to develop a clinical protocol combining LAI medications for the treatment of HIV and OUD, and subsequently evaluating the feasibility, acceptability, safety, and scalability of this novel, integrated delivery model in a single group, open pilot trial protocol.

## Methods

### Study design overview

The proposed project consists of two phases that will be conducted in an outpatient setting. Phase 1 includes in-depth individual interviews with PWH and have an OUD (*n* = 14–20) and in-depth interviews with key stakeholders in the fields of addiction medicine (*n* = 5–7), HIV care (*n* = 5–7), and pharmacy (*n* = 5–7). Research suggests that it is possible to achieve saturation with a sample size of 7–10 participants [44]. As such, we will aim to recruit a minimum of 7–10 patients in each subgroup (patients, stakeholders), however, we will continue recruitment until saturation of key themes is achieved. Phase 1 will culminate in the development of a clinical protocol to guide the delivery of combined LAI treatment for HIV and OUD and will be directly informed by the interview data. In Phase 2, the clinical protocol and implementation approach will be piloted with 40 PWH and an OUD. A sample size of 40 was selected to garner information about our study design as well as determine what modification may be needed to develop a larger, hypothesis testing, study in the future [45, 46]. Participants enrolled will first complete a baseline interview, followed by an evaluation with a physician to determine appropriateness for LAI (for HIV and OUD). Participants will then be scheduled to complete injection appointments to initiate CAP/RPV and XR-B. Follow-up interviews will occur at 1-, 3-, and 6-months post LAI injection to evaluate feasibility/acceptability and safety of the combined LAI treatment. Feedback from participants, study staff, and stakeholders will guide further refinement of the protocol and implementation approach for a future large-scale study. At the conclusion of Phase 2, we will have finalized the clinical protocol including recommendations for implementation. These data will be used to inform the development of a large-scale, fully-powered, Hybrid Type I Effectiveness-Implementation trial. At the time of this report, recruitment for Phase 1 is ongoing. This study is registered at ClinicalTrials.gov under protocol #NCT05991622. The study was registered during Phase 1, prior to the onset of the single-arm open pilot trial.

### Phase 1

Qualitative in-depth, individual interviews are currently being conducted with PWH who meet the criteria for a lifetime history of OUD, and key stakeholders relevant to addiction medicine and HIV care. Following informed consent, demographic and clinical data are obtained for participant interviews. Interviews are conducted by a trained Research Assistant either in-person in a private room or via videoconferencing (per participant preference). Interview guides are informed by the Adaptome, a framework developed to advance the science of intervention adaptation that highlights multiple dimensions to consider when attempting to optimize the fit of existing intervention(s) within a specific context [47]. Because the combined LAI treatment builds upon two existing interventions, the dimensions of the Adaptome are used to guide development of an integrated intervention delivery model. There are two separate interview guides—one developed for potential patients and the other developed for key stakeholders—they contain questions across several key areas: *service setting considerations* (e.g., who will deliver the intervention?, which setting(s) are optimal for intervention provision?, how will the intervention fit with existing service provision?, who will finance the intervention delivery?); *target audience considerations* (e.g., how will patients be identified for LAI receipt?, how will eligibility and appropriateness for LAI receipt be determined?); *mode of delivery considerations* (e.g., how many sessions should be required prior to and following injection receipt?, how long should the injection process take?, what education, if any, should be provided along with the injections?, what additional services are needed to ensure adequate support and treatment response?); and *cultural adaptations* (e.g., how might this delivery be modified to meet the needs of special populations?). Each interview lasts approximately 45–60 min and participants receive $50 for their time. Interviews are transcribed by a professional transcription agency, and reviewed for accuracy by the Research Assistant.

Trained Research Assistants will review and clean all transcripts prior to uploading them into QSR NVivo [11, 48], a qualitative data management and analysis software. A preliminary coding structure will be derived deductively from the interview topic guides, with specific subtype coding applied inductively as themes and repetitions emerge from the data. Two independent coders (research assistants trained in qualitative methods) will code each transcript. The coders, in conjunction with the Principal Investigator (PI; KJL), will develop a list of thematic codes arising from the data, which include a priori codes derived from the agendas as well as codes arising from emergent data [49, 50]. The coders will double-code all transcripts. The coders and PI will meet to review and discuss the code assignments and reconcile any discrepancies. After consensus is reached, a final master code will be entered into NVivo. Data analysis will be iterative using standard analysis techniques, including open coding, axial coding, marginal remarks, and memo-writing [49, 50]. Transcripts will be reviewed by the PI and other members of the study team, who will discuss transcripts to analyze themes, conduct subcode analyses and stratified analyses where indicated, and determine the implications of the data for protocol development. An audit trail of coding decisions and other aspects of analysis will be kept.

### Phase 2

Information garnered from Phase 1, including level of interest in LAI, will be used to develop a clinical protocol to guide the delivery of the combined LAI treatment. Interviews in Phase 1 will also explore barriers and facilitators to medication uptake, which will be addressed in the clinical protocol, with the goal of bolstering participation. Forty participants diagnosed with HIV and an OUD, and meeting the other study inclusion criteria (described below in “Participants” section), will be enrolled in an open pilot trial designed to examine metrics of feasibility, acceptability, and safety. Participants will complete a baseline interview, receive the combined LAI treatment (described below in “Combined LAI treatment for OUD and HIV” section), and complete follow-up assessments at 1-, 3-, and 6-months following initiation of injectable medication. Of the 40 participants, 24 will be invited to engage in qualitative interviews to assess the strengths and limitations of the clinical protocol and combined treatment as well as describe their reasons for LAI uptake or discontinuation; the 24 participants will be purposefully selected based on level of engagement. Specifically, we will conduct interviews with 12 participants with high levels of engagement in the clinical protocol and 12 participants with low engagement with the clinical protocol. We will also elicit feedback from clinic staff and other key stakeholders regarding the delivery of the clinical protocol and other implementation factors.

### Participants

In both phases, inclusion criteria for patients will reflect the requirements for initiating LAI ART and/or XR-B and are as follows: (1) 18–65 years of age; (2) HIV-1 infection, documented by any licensed rapid HIV test or HIV enzyme or chemiluminescence immunoassay; (3) current diagnosis of an OUD, moderate-severe, according to DSM-5; (4) not currently pregnant, breastfeeding, planning to become pregnant or breastfeed during the study period; (5) no coinfection of hepatitis B, or plans to get treated for hepatitis C during the study period; (6) amenable to starting injectable medications; (7) able to understand and speak English; 8) able and willing to provide written and verbal informed consent. Patients who are actively engaged in other MOUD regimens will still be considered if they are interested in transitioning to XR-B. Exclusion criteria include: (1) those that are not virologically suppressed (HIV-1 RNA > 50 copies/mL) given this aligns with the current FDA approval for administering CAB/RPV; (2) resistance to cabotegravir or rilpivirine; or (3) allergies, significant drug–drug interactions, or any other contraindications to the individual components of the medications. Interviews will continue until saturation is achieved. Inclusion criteria for key stakeholders are: (1) ≥ 18 years of age; (2) have experience working with populations diagnosed with either HIV, OUD, or a co-occurrence of both conditions; (3) have knowledge of medications used to treat HIV and OUD as defined by prior receipt of training in the provision of these medications; (4) fluent in English; and (5) are willing and able to provide informed consent.

### Study setting

#### The Miriam Hospital Immunology Center

The Miriam Hospital Immunology Center is in Providence, Rhode Island and is part of the larger Lifespan Healthcare System. The Immunology Center cares for nearly 90% of persons infected with HIV/AIDS who are in care in Rhode Island and also patients from nearby areas of Connecticut and Massachusetts. The Immunology Center currently serves over 1800 PWH, providing comprehensive HIV care including gynecological care, mental health support, substance use treatment with medication (primarily buprenorphine products), and case management [51]. Recent clinic data suggests that there are over 100 patients actively involved in treatment for OUD within the Immunology Center.

### Study procedures

The study team will work with clinic staff to inform patients of the potential research opportunity. Potential participants will also be informed of the study through social media, flyers, and mailings. Research personnel will approach potential participants to carefully explain all aspects of the study, obtain informed consent, and determine initial eligibility. All assessments and interviews will occur in a private room at the Miriam Hospital Immunology Center.

### Compensation and retention

To enhance likelihood of study retention, an increasing schedule of monetary compensation will be utilized. Participants will receive $40 for completion of the baseline interview, $40 for completion of assessments administered following the injection appointment, and $45, $50, and $60, respectively, for the 1-, 3-, and 6-month follow-up appointments. Additionally, the 25 participants who complete a qualitative interview at the end of the study will receive an additional $50.

## Intervention condition

### Combined LAI treatment for OUD and HIV

The specific timing, structure, and implementation approach for combined LAI treatment will be guided by the in-depth interviews conducted during Phase 1. However, the anticipated protocol for combined LAI treatment in the outpatient setting is described herein. Key treatment team members will include HIV Physicians, Addiction Medicine Physicians, Pharmacists, Adherence Nurses, Administering Nurses, and Pharmacy Liaisons. Additionally, given the high-risk and vulnerable nature of this population, it is expected that additional wrap-around services may be warranted such as access to a certified peer recovery specialist, social work/case management, and behavioral health counseling. These services are readily available through the Immunology Center and will be incorporated into the clinical protocol should this emerge as a recommendation based upon the qualitative data obtained in Phase 1.

#### Lead-in period

Participants will begin the clinical protocol with an initial evaluation with a physician to evaluate appropriateness for LAI formularies, discuss the advantages, disadvantages, safety, and efficacy of medication, provide education on the relationship between HIV and OUD and the impact on treatment, emphasize the importance of maintaining monthly injections, and answer any questions. At this appointment, patient who have not yet initiated buprenorphine will be prescribed sublingual buprenorphine for 7 days prior to initiating XR-B.

#### Initiating injectables

At the injection visit, an adherence nurse will administer the injections according to standard instructions and observe the participant for approximately 10 min to ensure there is no adverse reaction. The participant will be scheduled for a second injection appointment within 26–28 days, as well as a follow-up visit with the study physician. The treatment team will outreach the participant, via phone/text, in weekly intervals following the first injections to assess for tolerability, adverse effects, and desire to continue this course of treatment. The timing of each injection (XR-B and CAP/RPV) may not initially be concurrent, but the treatment team will work with patients to adjust the dosing to eventually achieve co-administration of both treatments. The XR-B is injected into the abdominal subcutaneous space, whereas the ART is injected into both gluteal muscles. The LAI medications will not be routinely provided as part of the study in an effort to reflect “real-world” conditions. An important component of the study will be to evaluate if the insurance process presents as a barrier to CAB/RPV and XR-B uptake.

#### Missed appointments/discontinuation

The treatment team will be in close contact with participants to encourage appropriate follow-up care and adherence to medication. If the patient is rescheduled within 35 days from last injection, no additional intervention is necessary. If, however, the ART injection is missed within the approved timeframe, oral ART will be resumed and reevaluation by a physician will be required prior to restarting the injections. A similar protocol will be followed for XR-B. That is, participants who do not return to the clinic within 6 weeks of the last injection, will be encouraged to resume sublingual buprenorphine for 7 days prior to restarting injectables. Participants wishing to discontinue LAI (ART or XR-B) will be encouraged to resume oral/sublingual therapies. To increase our ability to follow participants and encourage treatment adherence, permission will be requested from participants during the consent process to obtain additional contact information from close family members and friends to outreach in the event the participant does not present for scheduled appointments.

## Assessments (described in Table 1)

### Screening

The study screening will assess all inclusion and exclusion criteria. Self-reported HIV status will be confirmed with HIV test result extracted from participants’ medical record. Pregnancy tests will be performed on all people assigned female at birth, of childbearing age, without documented tubal ligation or hysterectomy. This will be completed as part of routine clinical care and verified via the medical record. Hepatitis B and C coinfection will similarly be determined through medical record review.

**Table 1:** Study measures

Domains	Instrument/variables	Assessments				
		Baseline	Injection	Post-Injection		
				1-month	3-month	6-month
Demographics	(PhenX) Demographics + structural determinants of health	**X**				
Feasibility/acceptability: % of participants: - Interested - Eligible - Consented - Initiated injectables - Retained in treatment Degree of: - Satisfaction - Treatment effectiveness	Medical record review study documentation					**X**
	Client satisfaction questionnaire-revised [70]					**X**
	Treatment effectiveness assessment			**X**	**X**	**X**
Safety: - HIV viral load - Adverse events	Medical record review	**X**	**X**	**X**	**X**	**X**
Secondary measures: - Substance use - HIV risk behavior - Treatment received - MOUD utilization - Quality of life	NIDA-modified ASSIST screener-lifetime	**X**				
	Current alcohol and other drug use (NESARC)	**X**	**X**	**X**	**X**	**X**
	Medical record review	**X**	**X**	**X**	**X**	**X**
	TCU HIV/AIDS risk assessment	**X**	**X**	**X**	**X**	**X**
	Treatment services review		**X**	**X**	**X**	**X**
	(PhenX) Quality of life	**X**	**X**	**X**	**X**	**X**
Implementation factors - Patient - Provider - Organization	Qualitative interview					**X**
	Acceptability of intervention measure					**X**
	Intervention appropriateness measure					**X**
	Feasibility of intervention measure					**X**
	Implementation climate					**X**
	Organizational readiness for implementation change					**X**

### Demographics

Using the PhenX toolkit [52], a web-based catalog of recommended measurement protocols, basic demographic data and social determinants of health will be collected at baseline to characterize the sample of participants.

### Feasibility/acceptability

At the conclusion of the study, information will be compiled about the percent of patients interested in the treatment, patient eligibility rate, consent rate, rate of recruitment, percent of participants who initiate injectables, follow-up completion rate (through 6 months), and reasons for refusal to evaluate the feasibility of conducting a subsequent larger scale study using this protocol. Additionally, study withdrawal rates and rates of discontinuation of injectables will be measured as indices of acceptability. Intervention acceptability and feasibility will also be assessed by asking each participant to complete the 8-item Client Satisfaction Questionnaire-Revised which has been extensively studied, has excellent internal consistency, reliability, and convergent validity, and has been shown to operate similarly across different racial and ethnic groups [53]. Self-report of engagement in treatment will be assessed with the 4-item Treatment Effectiveness Assessment [54], a measure with acceptable reliability and validity used in measuring treatment progress and recovery for substance use disorders including OUDs.

### Safety

To determine safety of the proposed intervention, HIV viral loads (and viral resistance as applicable)) will be measured at all timepoints. This lab work will be completed as part of usual care, and results will be extracted from the electronic medical record. The rate at which an adverse event occurs, as documented by the clinical team in the medical record and/or self-reported by the participant, will be assessed to further evaluate safety.

### Secondary measures

To inform how this treatment may impact relevant factors associated with HIV and OUD, other substance use will be measured through standard tools (National Epidemiologic Survey on Alcohol and Related Conditions; NESARC [55], NIDA-modified ASSIST [53]), as well as urine drug screens (collected via usual care and documented in the electronic medical record). HIV Risk Behavior will be assessed by the TCU HIV/AIDS Risk Assessment [56] and will serve as a measure of overall sex and drug risk behavior. The Treatment Services Review will be used to assess receipt of case management, psychiatric, peer recovery coaching, and other treatment services, including utilization of other medications for OUD (e.g., if a participant has transitioned to Methadone). At follow-up interviews, participants will be asked about services received since their previous interview. Overall quality of life will be assessed using the Quality-of-Life Enjoyment and Satisfaction Questionnaire – Short Form, a psychometrically sound shorter version of the original Quality of Life Enjoyment and Satisfaction Questionnaire. It consists of a self-reported 16-item questionnaire that has shown internal consistency, test–retest reliability, and convergent and criterion validity (80% sensitivity, 100% specificity) [57]. This data will be used to inform a subsequent larger scale study using this clinical protocol.

### Implementation factors

Throughout the open pilot trial, we will assess organizational-, patient-, and provider-related factors associated with future implementation. *Patient*. In addition to the feasibility, acceptability, and safety measures described above, 24 purposively sampled participants will be asked to complete a qualitative interview in which they will be asked to reflect on factors that impacted their use or non-use of combined LAI treatment. *Provider:* The Acceptability of Intervention Measure (AIM), Intervention Appropriateness Measure (IAM), & Feasibility of Intervention Measure (FIM) will be employed [58]. Each of these measures contains four items assessing implementation outcomes that are often considered “leading indicators” of implementation success [59]. *Organizational—*We will explore factors related to the implementation climate and organizational readiness, two measures associated with an organization’s inner setting that have been shown to influence implementation. Implementation climate will be assessed by a 6-item measure of provider perceptions regarding the extent to which the innovation being implemented (i.e., combined LAI) is expected, supported, and rewarded within their organization. Each item is scored on a 5-point Likert scale. This well-validated, brief measure was developed by Jacobs and colleagues, and following published guidelines, we calculate a scale mean for each staff member. Organizational readiness will be assessed by the Organizational Readiness for Implementing Change measure developed by Shea and colleagues. This 12-item scale will be administered to all participating staff. Each item assesses provider perceptions regarding the extent to which their organization has the level of skill, training, time, and resources necessary to implement the combined LAI protocol. Qualitative Interviews—at the conclusion of the study, we will conduct qualitative interviews with providers and other clinic staff to explore other implementation barriers and facilitators to combined LAI treatment. Consistent with the guidelines for measure administration, the term “innovation” has been adapted to reference the combined LAI intervention.

## Data analysis

Analyses for this pilot study will have the primary goal of establishing feasibility, acceptability, and safety of concurrently administering LAI medications for HIV and OUD. Feasibility will be assessed via a range of indicators, including the percent of patients interested in the treatment, patient eligibility rate, consent rate, rate of recruitment, percent of participants who initiate injectables, follow-up completion rate (through 6 months), study attrition, and rates of medication discontinuation. Acceptability will be assessed by calculating mean scores for the Client Satisfaction Questionnaire and Treatment Engagement Assessment. Feasibility and acceptability will be reflected by: > 75% of eligible participants providing consent; > 75% of participants who consent initiating both injections; > 80% of participants retained at 6 months; change of > 8 points from baseline to 6-month follow-up on the Treatment Engagement Assessment; and high satisfaction indexed by > 24 on the Client Satisfaction Questionnaire. Safety will be evaluated by analyzing HIV viral loads, and the development of any virologic resistance across the study period, as well as the incidence of adverse events.

Secondary analyses for this study will examine the implementation potential of the integrated intervention. The qualitative and quantitative data obtained in Phase 2 of the study will be integrated via triangulation. That is, the two data sets will be compared to determine whether they converge, offer complimentary information, or contradict each other to offer a more nuanced understanding of the research question [60].

## Discussion

The co-occurrence of HIV and OUD is associated with numerous adverse health outcomes including increased risk of morbidity, mortality, and HIV transmission [8–10, 16, 17]. Although evidenced-based treatments are available to address these conditions, many PWH who have OUD struggle to maintain adherence to their medications, given the burden of daily oral administration as well as social and structural factors that create barriers to care engagement (e.g., substance use-related stigma, transportation barriers [61, 62]). This is notable in that PWH who have an OUD are both particularly vulnerable for fatal overdose and are less likely to achieve HIV-viral suppression, contributing to higher mortality rates among this population and potential risk for onward HIV transmission [8–10, 15–17]. As such, there have been recent calls to develop interventions to address the unique challenges associated with the intertwined HIV and OUD epidemics [31, 32].

Recent pharmacological advancements have led to the development and approval of LAI medications for both HIV and OUD. These medications have the potential to significantly improve adherence, lead to better control of both diseases, and reduce mortality rates for PWH with OUD [35–37, 42, 43]. While combining these two treatments into a single point of care holds promise, currently little is known about whether offering integrated treatment of CAB/RPV and XR-B is feasible, acceptable, or safe for this vulnerable population. Further, to maximize use and impact of this integrated treatment package, formative evaluation with the target populations (e.g., patients who might engage in this treatment; providers who might offer/deliver this treatment) is critically needed to guide the delivery model. Accordingly, this study aims to fill gaps in prior research by first conducting formative research to develop a clinical protocol combining LAI medications for the treatment of HIV and OUD, and then evaluating the feasibility, acceptability, safety, and scalability of this novel integrated delivery model.

At the conclusion of this study, our goals are to (a) develop a treatment protocol with input from the target population, (b) evaluate the feasibility/acceptability/safety of the proposed protocol, and (c) assess relevant factors to optimize implementation potential. Results of this study will be used to inform the development of a large-scale, fully-powered, Hybrid Type I Effectiveness-Implementation trial. The assessment of patient-, provider-, and organizational-factors that may impact future implementation potential for combined LAI treatment will yield valuable information to inform future intervention development efforts, and will help us to accelerate the translation of the integrated intervention to clinical settings.

This work has the potential to improve the treatment and prevention of HIV infection among a population where substance use is a significant contributing factor, by integrating treatment for OUD and HIV infection. If successful, this study has high clinical and public health significance by developing and testing the preliminary efficacy of a treatment protocol which meaningfully combines the two LAI treatments to improve clinical outcomes for PWH with OUD. The knowledge gained through this study has the potential to reduce the lag time between research discovery and clinical uptake in clinical settings, and may increase our knowledge of how to promote the uptake of other long-acting medications such as long-acting PrEP.

The current study is not without limitations. First, the sample will be comprised of English-speaking participants, which limits generalizability to other racial/ethnic groups. Second, given current FDA guidelines, only participants with suppressed viral loads will be enrolled. However, there is emerging data suggesting the potential for CAP/RPV among persons without suppressed viral loads. Thus, investigating the use of CAP/RPV among persons without viral suppression is a priority for future research.

## Data Availability

Not applicable.

## Abbreviations

PWH: People living with HIV
OUD: Opioid use disorder
ART: Antiretroviral therapy
LAI: Long-acting injectable
CAB/RPV: Cabotegravir/rilpivirine
XR-B: Extended-release buprenorphine
IDIs: In-depth individual interviews
